# Assessment of menstrual hygiene management practice and associated factors among prisoners in South Nation Nationalities and peoples region, Ethiopia

**DOI:** 10.1016/j.heliyon.2023.e16224

**Published:** 2023-05-25

**Authors:** Sindu Degefu, Aster Tadesse, Kidist Ashagir, Elias Ezo

**Affiliations:** a Department of Nursing College of Medicine and Health Science, Wolaita Sodo University, Ethiopia; b Department of Nursing College of Health Sciences, Debre Markos University, Ethiopia; c Department of Comprehensive Nursing School of Nursing College of Medicine and Health Science, Wachemo University, Ethiopia

**Keywords:** Menstrual hygiene management practice, Women, Prison institution

## Abstract

**Introduction:**

Menstrual hygiene management practice is the requirements and necessities; such as the use of a sanitary pad or clean and mushy absorbents, sufficient washing of the genital area, proper disposal of the used absorbents, and other special needs for the women during menstruation.

**Objective:**

To assess menstrual hygiene management practice and associated factors among prisoners in south Nation Nationalities and Peoples Region, Ethiopia, 2022.

**Methods:**

An institution-based cross-sectional study was conducted from May 1 to July 30/2022. The total sample size was 605 and a simple random sampling technique was used to select prison institutions and women. The data were collected through face-to-face interviews. Data were entered using Epi data 4.6 version and analyzed by using SPSS version 26 software. Multicollinearity was checked and the goodness of fit test was done by using the Hosmer Lemeshow model of goodness fit test. Univariate analysis was done and variables with p value less than 0.25 were taken to bivariate logistic regression analysis. Adjusted odds ratio with the 95% confidence interval was considered and statistical significance was at a p-value less than 0.05 in bivariate logistic regression analysis.

**Result:**

The prevalence of menstrual hygiene management practice was 50.6% (95% CI 47.3–54.4). Age 19–29 years old [AOR: 5.03, 95% CI 1.73–14.62], educational status; not formally educated [AOR: 0.05, 95% CI 0.02–0.13], educational status; primary level [AOR: 0.17, 95% CI 0.07–0.39], previous occupation; student [AOR: 2.56, 95% CI 1.06–6.21], previous occupation; a private employee [AOR: 4.11, 95% CI 1.48–11.42], previous occupation; government employee [AOR: 3.46, 95% CI 1.18–10.14], absence of support from family [AOR: 0.14, 95% CI 0.08–0.24] and absence of work engagement in prison [AOR: 0.44, 95% CI 0.25–0.78] were associated with MHMP.

**Conclusion:**

In this study, about five from ten women in prison practice menstrual hygiene management. Age, educational status, previous occupation, support from family, and work engagement in prison were important risk factors for MHMP. Therefore, support from family and engaging the women to work in prison institutions may increase the MHMP in prison institutions.

## 1 Introduction

Menstruation is a periodic shedding of the inner lining of the uterus through the vagina along with blood under the control of hormones of a hypothalamus-pituitary-ovarian axis [1]. Although it is a physiological process, menstruation is still surrounded by social taboos, supernatural beliefs, misconceptions, and malpractices which is very challenging for women in developing countries [[2], [3], [4], [5]].

Menstrual hygiene management practice (MHMP) is the requirements and necessities; such as the use of a sanitary pad or clean and mushy absorbents, sufficient washing of the genital area, proper disposal of the used absorbents, and other special needs for the women during menstruation [1,2,4]. In a woman's life, hygienic practice during menstruation is very crucial which prevents her from adverse health outcomes [4,6]. In low and middle-income countries, many girls are not able to manage their menses and associate hygiene with ease and dignity [2,[7], [8], [9]]. Women living in prisons are often subject to harsh and non-dignified treatment and many women in prison experience a lack of privacy during menstrual bleeding [1,6,7]. Globally, there are half a million female prisoners [10,11].

The prison environment does not always consider the specific needs of the women as the availability of hygiene requirements [7,11,12]. Due to the lack of availability of menstrual hygiene requirements, women who menstruate are further subjected to humiliation and resort to unhygienic means of menstrual management which can be dangerous to their future health status [5,11,[13], [14], [15], [16], [17], [18], [19]].

Poor MHMP is common among adolescent girls and women in poor communities whether in prison or not [5,13,14,20]. Female prisoners face healthcare challenges related to menstruation [14]. Incarcerated women as human beings have the right to a standard of living adequate for a healthy life [21]. However, evidence shows that the basic human rights of incarcerated people are in creditable still [22].

Although the Ethiopian government with various national and international sectors is using efforts, the MHMP was neglected [18,20,23]. Due to this, women prisoners have no women-specific health and sanitary care services. In contrast, there are poor medical services, inadequate toilets and bathrooms, and inaccessible sanitary pads [12,24]. In Ethiopia, the literature indicates that researchers also focused on school girls [25] and there is no previous study conducted on MHMP among prisoners in Ethiopia. Therefore, this study assessed the MHMP and associated factors among prisoners in South Nation Nationalities and Peoples Region, Ethiopia.

## 2 Methods and materials

### 2.1 Study area and period

The study was conducted in South Nation Nationalities and Peoples Region, Ethiopia. The region is one of the regions in Ethiopia, in which peoples with different nations, languages, cultures, and customs live together in tolerance. There are fifteen zonal prison institutions in the region. There are around 11,000–14, 000 prisoners in the region and 100,000–120,000 in Ethiopia, from which approximately 4–9% are women prisoners. The study was conducted from May 1 to July 30/2022.

#### 2.1.1 Study design

An institution (prison)-based cross-sectional study was conducted.

#### 2.1.2 Source population

All women prisoners in South Nation Nationalities and Peoples Region.

#### 2.1.3 Study population

All selected women prisoners in South Nation Nationalities and Peoples Region.

#### 2.1.4 Eligibility criteria

Women imprisoned in selected zonal prison institutions of South Nation Nationalities and Peoples Region who had at least one menstruation in the prison institution were included, however, severely ill women that cannot give a response during data collection period were excluded.

### 2.2 Sample size determination

The sample size was determined using the single population proportion formula. The assumption of 50% population proportion was used as no previous study in the country, 95% confidence level, and 5% margin of error as follows:

$$\frac{{\left(\frac{za}{2}\right)}^{2}p\left(1-p\right)}{d^{2}}=\frac{{\left(1.96\right)}^{2}0.5\left(1-0.5\right)}{{\left(0.05\right)}^{2}}=\frac{{\left(1.96\right)}^{2}0.5\left(0.5\right)}{0.0025}=384$$

By considering the 5% non-response rate and 1.5 design effect, the total sample size was 605.

### 2.3 Sampling technique and sampling procedure

**Fig. 1:**
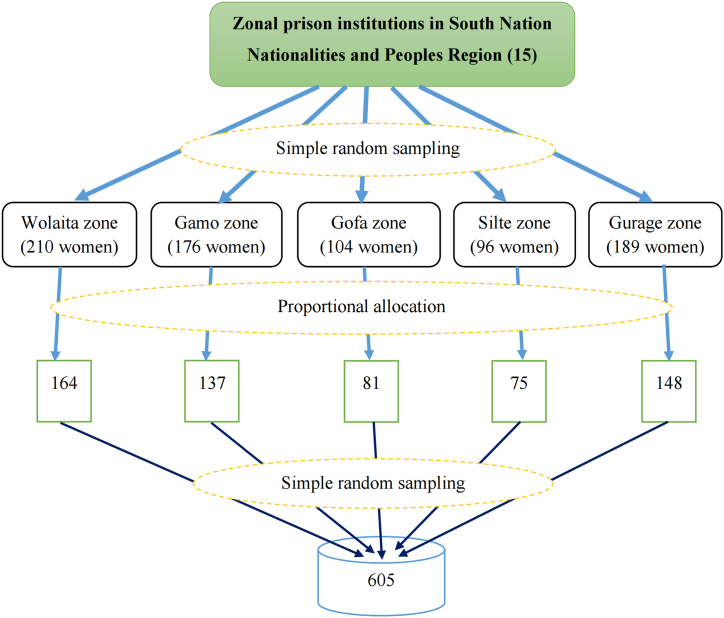
Diagrammatic showing sampling procedure of MHMP and associated factors among prisoners in south Nation Nationalities and Peoples Region, Ethiopia, 2022.

From the fifteen zonal prison institutions in the region, five zonal prison institutions (Wolaita, Gamo, Gofa, Silte, and Gurage) were included in the study by using a simple random sampling technique. Then, the total number of women prisoners in each prison institution was identified and allocated according to the sample size. Finally, the women for the interview were selected by using a simple random sampling technique. (Fig. 1).

### 2.4 Study variables

#### 2.4.1 Dependent variable

Menstrual hygiene management practice

### 2.5 Independent variables

#### 2.5.1 Sociodemographic factors

Age, educational status, previous residence, religion, marital status, period of confinement in prison, previous occupation, family support, and work engagement in prison.

#### 2.5.2 Knowledge-related factors

Knowledge of menses and discussion on menstruation.

#### 2.5.3 Prison facility-related factors

Latrine access, females' and males' toilets in opposite directions, toilets kept locked inside, separate rooms to manage menstruation and healthcare services, and free prison sanitary pad access.

### 2.6 Operational definition

#### 2.6.1 Prisoner women

Women kept in prison as a punishment for crimes they have committed or while awaiting their trial.

#### 2.6.2 Knowledge about menstrual hygiene

Measured using eleven [11] questions that ask about knowledge of menstrual hygiene management practice (what menstruation is, cause of menstruation, the source of menstrual blood, normal duration of menstrual bleeding, normal interval of the menstrual cycle, know that menstruation is up to menopause, foul-smelling during menstruation, is menstrual blood hygienic, heard about menstruation before menarche, sanitary pads available in the market, freely discuss menstruation issues with your parents or friends). For each question, a correct answer scored one [1] point while wrong and do not know answers scored two [2] point. Then, the mean 6.42 was used as a cut-off point to categorize as good knowledge (if scored mean and above) and poor knowledge (if scored below mean) [26,27].

#### 2.6.3 Menstrual hygiene management practice

It is the practice of menstrual hygiene management practice during menstruation in a prison institution. The women were asked whether they used sanitary material during menstruation. Those who answered “yes” were further asked for the type sanitary material they used, and also those who answered “no” were asked for the reason why they did not use. Following this, the women asked whether they wash genitalia during menstruation. Those who answered “yes” again asked for the material used addition to water for washing and the frequency of washing. At third level, the women were asked whether they take bath during menstruation, and frequency of bath was asked by those women who answered “yes”. At fourth level, whether the women change sanitary material during menstruation asked and for those women who scored “yes” the frequency per day was asked. After this, the women were asked whether they dispose the used sanitary material. Those women who answered “yes” were farther asked for the process how they dispose the used disposable and reusable material, how wash the reusable material and how they keep for drying after wash.

However, various literatures used different for this outcome [[26], [27], [28], [29], [30], [31], [32]]. Due to this, the authors of this research used the five [5] main questions to measure menstrual hygiene management practice. The calculation was done using five [5] main questions asking about menstrual hygiene management practice in the prison institution, that were (use sanitary material during menstruation, wash genitalia during menstruation, take bath during menstruation, change sanitary material during menstruation, and dispose used the used sanitary material). Each question had two alternatives (1, no and 2, yes). Then, the individual response of each question was recoded, and categorized as good MHMP (if scored mean and above) and poor MHMP (if scored below mean), which was 5.481.

### 2.7 Data collection tool and procedure

A structured tool was prepared in English by adapting from various related literature [13,15,19,26,33]. The data were collected through face-to-face interviews with the women in prison. The data collection process was carried out by five clinical nurses and five experienced public health officers supervised the collection procedure.

### 2.8 Data quality assurance

The tool was translated to Amharic (the local language of Ethiopia), before data collection and retranslated back to English after data collection. Both translations were done by language expertise. One day of training was given to data collectors and supervisors. A pretest was done on 5% (30 women) of the total sample size in the Hadiya zone prison. Based on the pre-test result, all the relevant corrections were made. The reliability of the questionnaire was assessed and the reliability index (Cronbach's Alpha), which was 0.79. Each questionnaire was reviewed and checked for completeness.

### 2.9 Statistical analysis

Data were entered by using Epi data 4.6 version and analyzed by using SPSS version 26 software. The descriptive findings were presented by using mean, standard deviation, frequency tables, graphs, and percentages. The variable inflation factor (VIF > 10) was used to test multicollinearity between independent variables. The goodness of fit test was done by using the Hosmer Lemeshow model of goodness fit test. To determine the independent factors associated with the menstrual hygiene management practice, univariate analysis was done and variables with p value less than 0.25 were taken to bivariate logistic regression analysis. Adjusted odds ratio with the 95% confidence interval was considered and statistical significance was at a p-value less than 0.05 in bivariate logistic regression analysis.

## 3 Result

### 3.1 Sociodemographic characteristics

Six hundred five women gave a response that made the overall response rate 100%. More than half, 353 (58.3%) of women were in the age group of 19–29 years old, and 180 (29.8%) of women were not formally educated. The residence of 306 (50.6%) women was urban and more than half, 333 (55.0%) of women were a follower of the protestant religion. About 278 (46.0%) of women were married and the previous occupation of 162 (26.8%) women was a daily laborer. The majority, 541 (89.4%) of women had a duration of imprisonment of fewer than five years. Almost half, 305 (50.4%) of women had no support from family, and 319 (52.7%) of women were work engagement in prison (Table 1).

**Table 1:** Sociodemographic characteristics of prisoners in south Nation Nationalities and Peoples Region, Ethiopia, 2022.

Variable (n = 605)	Category	Frequency	Percent
Age of women	19–29 years old	353	58.3%
	30–39 years old	214	35.4%
	40–49 years old	38	6.3%
Educational status	Not formally educated	180	29.8%
	Primary level	161	26.6%
	Secondary level	174	28.8%
	College and above	90	14.9%
Previous residence	Urban	306	50.6%
	Rural	299	49.4%
Religion	Orthodox	155	25.6%
	Muslim	108	17.9%
	Protestant	333	55.0%
	Catholic	9	1.5%
Marital status	Single	227	37.5%
	Divorced	58	9.6%
	Widowed	42	6.9%
	Married	278	46.0%
Previous occupation	Housewife	128	21.2%
	Student	121	20.0%
	Daily laborer	162	26.8%
	Merchant	71	11.7%
	Private employee	50	8.3%
	Government employee	73	12.1%
Duration of imprisonment	Less than five years	541	89.4%
	Five to ten years	59	9.8%
	Greater than ten years	5	0.8%
Support from family	Yes	300	49.6%
	No	305	50.4%
Type of support (n = 300)	Material	53	17.7%
	Financial	14	4.7%
	Both	233	77.7%
Work engagement in prison	Yes	286	47.3%
	No	319	52.7%

### 3.2 Knowledge of women about MHMP

**Fig. 2:**
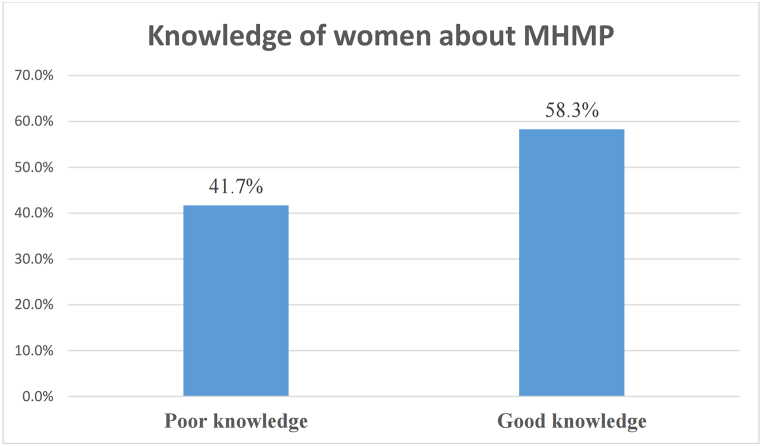
Knowledge of prisoners about MHMP in south Nation Nationalities and Peoples Region, Ethiopia, 2022.

Out of 605 prisoners, 252 (41.7%) had poor knowledge and 353 (58.3%) had good knowledge (Fig. 2).

### 3.3 Prison facility-related characteristics

The prisons had an open toilet in opposite direction to men's toilets, even though there was no lock inside and water access. All prisons had no sanitary pad access for a free and separate room to manage menstruation and they had healthcare services, but, were not equipped well.

### 3.4 Prevalence of MHMP

**Fig. 3:**
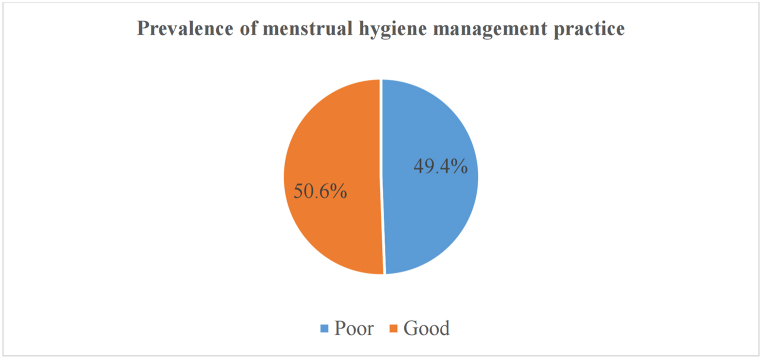
The prevalence of MHMP in south Nation Nationalities and Peoples Region, Ethiopia, 2022.

In this study, 299 (49.4%) of prisoners had poor and 306 (50.6%) had good MHMP (Fig. 3).

### 3.5 Prevalence of MHMP regarding the previous residence

**Fig. 4:**
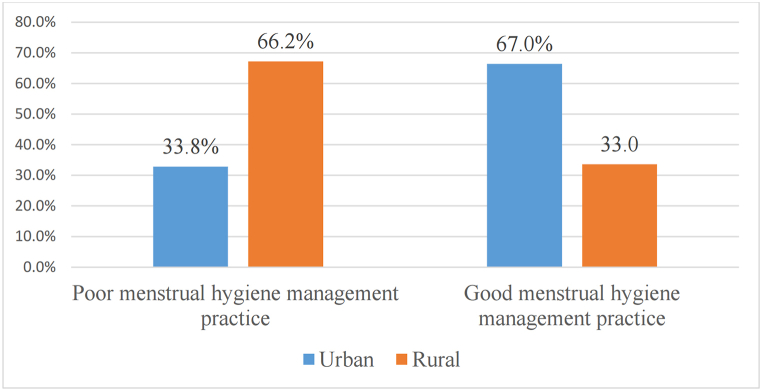
The prevalence of MHMP regarding the previous residence in south Nation Nationalities and Peoples Region, Ethiopia, 2022.

Of the 605 women prisoners, 10 (33.8%) of urban and 198 (66.2%) of rural previous residence women had poor MHMP, however, 205 (67.0%) of urban and 101 (33.0%) of rural previous residence women had good MHMP (Fig. 4).

### 3.6 Prevalence of MHMP regarding family support

**Fig. 5:**
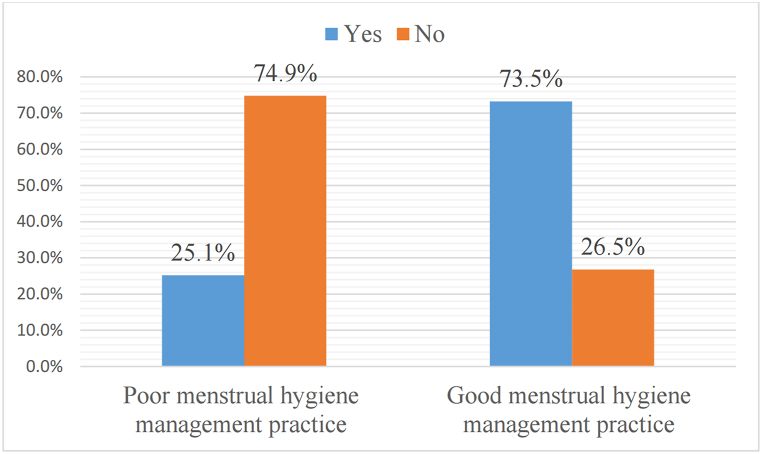
The prevalence of MHMP regarding family support in south Nation Nationalities and Peoples Region, Ethiopia, 2022.

In this study, 75 (25.1%) of women who have family support and 224 (74.9%) of women who have no family support had poor MHMP, on the other hand, 225 (73.5%) of women who have family support and 81 (26.5%) of women who have no family support had good MHMP (Fig. 5).

### 3.7 Prevalence of MHMP regarding knowledge

**Fig. 6:**
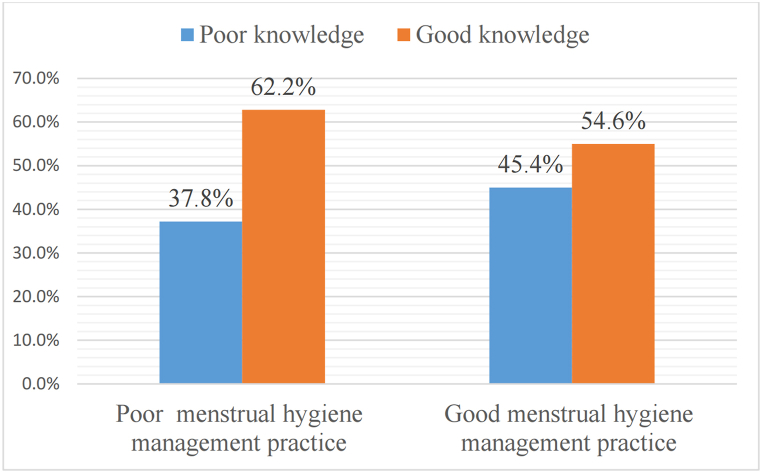
The prevalence of MHMP regarding knowledge in south Nation Nationalities and Peoples Region, Ethiopia, 2022.

Of the 605 women, 113 (37.8%) of poor knowledge and 186 (62.2%) of good knowledge had poor MHMP, whereas, 139 (45.4%) of poor knowledge and 167 (54.6%) of good knowledge had good MHMP (Fig. 6).

### 3.8 Factors associated with MHMP

In univariate analysis, age, educational status, previous residence, previous occupation, support from family, and work engagement in prison were associated with MHMP. Whereas, in bivariate logistic regression analysis; age 19–29 years old [AOR: 5.03, 95% CI 1.73–14.62], educational status; not formally educated [AOR: 0.05, 95% CI 0.02–0.13], educational status; primary level [AOR: 0.17, 95% CI 0.07–0.39], previous occupation; student [AOR: 2.56, 95% CI 1.06–6.21], previous occupation; a private employee [AOR: 4.11, 95% CI 1.48–11.42], previous occupation; government employee [AOR: 3.46, 95% CI 1.18–10.14], absence of support from family [AOR: 0.14, 95% CI 0.08–0.24] and absence of work engagement in prison [AOR: 0.44, 95% CI 0.25–0.78] were associated with MHMP. (Table 2).

**Table 2:** Univariate and bivariate factors associated with MHMP of prisoners in south Nation Nationalities and Peoples Region, Ethiopia, 2022.

Variable (n = 605)	Category	MHMP		COR (95% CI)	AOR (95% CI)	P value
		Poor	Good			
Age	19–29 years old	150 (24.8%)	203 (33.6%)	2.93 (1.43–5.99)	5.03 (1.73–14.62)	0.003*
	30–39 years old	123 (20.3%)	91 (15.0%)	1.60 (0.77–3.35)	2.74 (0.96–7.83)	0.061
	40–49 years old	26 (4.3%)	12 (2.0%)	1	1	
Educational status	Not formally educated	159 (26.3%)	21 (3.5%)	0.02 (0.01–0.04)	0.05 (0.02–0.13)	0.000*
	Primary level	101 (16.7%)	60 (9.9%)	0.09 (0.05–0.18)	0.17 (0.07–0.39)	0.000*
	Secondary level	27 (4.5%)	147 (24.3%)	0.84 (0.40–1.74)	0.83 (0.36–1.94)	0.672
	College and above	12 (2.0%)	78 (12.9%)	1	1	
Previous residence	Urban	101 (16.7%)	205 (33.9%)	1	1	
	Rural	198 (32.7%)	101 (16.7%)	0.25 (0.18–0.35)	0.92 (0.54–1.56)	0.757
Previous occupation	Housewife	57 (9.4%)	71 (11.7%)	1	1	
	Student	42 (6.9%)	79 (13.1%)	3.81 (2.02–7.19)	2.56 (1.06–6.21)	0.038*
	Daily laborer	108 (17.9%)	54 (8.9%)	5.75 (2.99–11.02)	1.56 (0.64–3.96)	0.323
	Merchant	27 (4.5%)	44 (7.3%)	1.53 (0.82–2.85)	0.90 (0.39–2.05)	0.802
	Private employee	10 (1.7%)	40 (6.6%)	4.98 (2.43–10.19)	4.11 (1.48–11.42)	0.007*
	Government employee	55 (9.1%)	18 (3.0%)	12.22 (5.10–29.28)	3.46 (1.18–10.14)	0.024*
Support from family	Yes	75 (12.4%)	225 (37.2%)	1	1	
	No	224 (37.0%)	81 (13.4%)	0.12 (0.08–0.17)	0.14 (0.08–0.24)	0.000*
Work engagement in prison	Yes	67 (11.1%)	219 (36.2%)	1	1	
	No	232 (38.3%)	87 (14.4%)	0.12 (0.08–0.17)	0.44 (0.25–0.78)	0.005*

**Abbreviations:** MHMP: menstrual hygiene management practice; COR: Crude Odds Ratio; AOR: Adjusted Odds Ratio: CI: Confidence Interval.

**Hint**: “*” = p < 0.05 statistically associated; “1” = reference group.

## 4 Discussion

The prevalence of MHMP was 50.6% (95% C·I 47.3–54.4). It was lower than studies conducted in Adama, Ethiopia 57% [6], Harari, Ethiopia 55.8% [34], Mehal Meda, Ethiopia 90.9% [35], Egypt 90% [36], and Nepal 67% [37]. It was in line with studies done in Ambo, Ethiopia 47.3% [38] and northeastern Ethiopia 53.9% [33]. However, it was higher than studies conducted in Wegera district schools, northwest Ethiopia 29.8% [26], Nigeria 25% [39], and Uganda 9.5% [40]. This difference might be due to variation in study institution (most researches were on high school girls), sample size variation, the age of participants, (most high school girls are youth), educational status of the study population (high school girls), and the prison institution setting, variations in sociodemographic differences among the study areas and population differences.

In this study, the age of women was statistically associated with MHMP. Women aged 19–29 years old were 5.03 times more likely to manage menstrual hygiene compared to women aged 40–49 years old. This was similar to other studies conducted in various high schools in Ethiopia [38,41]. This can be explained as young age groups having more access to information management and also they are more exposed to education and various Media as they are in the active age group. Health education including MHMP is started in schools as a club for students in recent years, as a result, the young age group women could have had the chance to gain information from their schools that might increase their capacity to manage menstrual hygiene.

Educational status has become a significant statistical factor for the MHMP of women in prison institutions. Women who were not formally educated were 95% less likely to manage menstrual hygiene compared to women whose educational status was college and above. And also women whose educational status is primary level were 83% less likely to manage menstrual hygiene compared to women whose educational status was college and above. It was with other studies conducted in Bahir Dar, Ethiopia [42] and Kathmandu prison, Nepal [43]. The possible explanation for this might be that educated women had a chance to prior knowledge and exposure in their schools about MHMP. So far, it is known that when women's educational level advances, they have more access to information about MHMP.

In this study, the previous occupation was identified as a significant factor for the MHMP of women in prison institutions. Women whose previous occupation was a student were 2.56 times more likely to manage menstrual hygiene compared to women whose occupation was a housewife. Similarly, women whose previous occupation was private employees were 4.11 times more likely to manage menstrual hygiene compared to women whose occupation was a housewife. Also, women whose previous occupation was government employee were 3.46 times more likely to manage menstrual hygiene compared to women whose occupation was a housewife. This might be related to being away from information and thereby less exposure to information about the MHMP. On the other hand, the student, private employees, and government employees were near to information as they stay with different women from various places. Additionally, these women were more exposed to educational levels than housewives. By nature, women keep their beauty more when going out of the home, however, this is less for housewives and their previous experience can also affect them accordingly in the prison.

In this study, support from family was statistically associated with MHMP. Women who had no support from family were 86% less likely to manage menstrual hygiene compared to women who had support from family. This was supported by other studies conducted in high schools in Ethiopia [6,33,44]. As the prison institution has no free sanitary pad access, those who had financial support can buy the requirements for MHMP including sanitary pads, those who had material support can have an opportunity that the material will be sanitary pads or they can interchange as necessary and those who had both financial and material support have various opportunities to gain the requirements and thereby MHMP.

Work engagement in prison was revealed as a significant statistical factor for MHMP of women in prison institutions. Women who had no work engagement in prison were 56% less likely to manage menstrual hygiene compared to women who had work engagement in prison. This might be explained as that it is known that if there was work, there would be money, and also due to the work, the women can be exposed to information, and manage their health status including MHMP. On contrary, those women not engaged in work and have no money stay in their rooms and thereby being far from information and the requirements needed for MHMP in prison institutions.

### 4.1 Limitations of the study

The study could be prone to social desirability and recall bias.

## 5 Conclusion

In this study, about five from ten women in prison practice menstrual hygiene management. Age, educational status, previous occupation, support from family, and work engagement in prison were important risk factors for MHMP in prison institutions. Therefore, support from family and engaging the women to work in prison institutions may increase the MHMP in prison institutions.

## Ethical consideration

Ethical clearance was obtained from Debre Markos University research ethical approval committee (DMU/HMRCS/190/2014) and given to the South Nation Nationalities and Peoples Region prison bureau. Next, a permission letter was sent from the bureau to each zonal prison institution.

## Author contribution

Sindu Degefu, Elias Ezo, and Kidist Ashagir: conceived and designed the experiments; Sindu Degefu performed the experiment. Sindu Degefu, Elias Ezo, and Aster Tadesse: analyzed and interpreted the data. Sindu Degefu, Aster Tadesse, and Kidist Ashagir: contributed reagents, materials, analysis tools or data; Elias Ezo: wrote the paper.

## Funding

The funding for this research was gained from Debre Markos University.

## Availability of data

The data are available upon secure and reasonable request.

## Informed consent

Written informed consent was obtained from each participant, and the information obtained from them would not have been disclosed. Coding was used to eliminate names and other personal identification of respondents to ensure anonymity, privacy, and confidentiality. Thoroughly, our research passed required the principles of the declaration of the Helsinki General Assembly, Seoul, Korea, and October 2008.

## Declaration of competing interest

The authors declare that they have no known competing financial interests or personal relationships that could have appeared to influence the work reported in this paper.
